# Extracorporeal Shock Wave Treatment (ESWT) enhances the *in vitro*-induced differentiation of human tendon-derived stem/progenitor cells (hTSPCs)

**DOI:** 10.18632/oncotarget.7064

**Published:** 2016-01-28

**Authors:** Laura Leone, Salvatore Raffa, Mario Vetrano, Danilo Ranieri, Florence Malisan, Cristina Scrofani, Maria Chiara Vulpiani, Andrea Ferretti, Maria Rosaria Torrisi, Vincenzo Visco

**Affiliations:** ^1^ Department of Clinical and Molecular Medicine, Faculty of Medicine and Psychology, “Sapienza” University of Rome, Rome, Italy; ^2^ Department of Orthopedics and Traumatology, Sant'Andrea Hospital, Faculty of Medicine and Psychology, “Sapienza” University of Rome, Rome, Italy; ^3^ Department of Biomedicine and Prevention, Laboratory of Signal Transduction, “Tor Vergata” University of Rome, Rome, Italy; ^4^ Cellular Diagnostics Unit, Sant'Andrea Hospital, Rome, Italy

**Keywords:** differentiation, tendon, stem cells, healing, ESWT, Pathology Section

## Abstract

Extracorporeal shock wave therapy (ESWT) is a non-invasive and innovative technology for the management of specific tendinopathies. In order to elucidate the ESWT-mediated clinical benefits, human Tendon-derived Stem/Progenitor cells (hTSPCs) explanted from 5 healthy semitendinosus (ST) and 5 ruptured Achilles (AT) tendons were established. While hTSPCs from the two groups showed similar proliferation rates and stem cell surface marker profiles, we found that the clonogenic potential was maintained only in cells derived from healthy donors. Interestingly, ESWT significantly accelerated hTSPCs differentiation, suggesting that the clinical benefits of ESWT may be ascribed to increased efficiency of tendon repair after injury.

## INTRODUCTION

The main cellular component of the human tendon consists of tenocytes that represent only the 5% of the normal tissue volume and are responsible for its maintenance and repair [1]. However, histological findings of injured tendons showed the presence of cells characterized by several phenotypes, suggesting that a possible multilineage differentiation potential may be involved either in tissue repair or in pathophysiology of such diseases, frequently characterized by fatty degeneration and chondroid calcification [2, 3]. On the other hand, the recovery of a normal function of injured tendons represents an ambitious goal in this field and various cell-based therapies with regenerative purpose for tendon-related pathologies have been recently performed [4-6]. The replacement of injured tenocytes and the restoration of a normal tissue homeostasis have been efficiently obtained by the injection of mesenchymal stem cells (MSCs) in the area of damaged tendons [5, 7].

Pathophysiology of tendinopathy, a degenerative disorder of the tendons, is still unclear and a better understanding of the cellular mechanisms involved in the healing process occurring in this pathology could strongly improve its therapeutic management [2]. The tendon healing is a multistep process involving inflammatory cells, cytokine release, extracellular matrix (ECM) remodelling and neoangiogenesis. In fact, tendinopathies can also be treated with autologous Platelet-rich plasma (PRP), containing large amounts of growth factors, some of them favoring tendon healing by cell recruitment and differentiation, while its clinical effectiveness remains matter of debate [8-10].

ESWT is an alternative treatment of some chronic tendinopathies. In order to explain its clinical benefits, we recently demonstrated that ESWT enhances cell proliferation, migration and secretory activity of human primary cultured tenocytes [11-13]. Previous reports suggested that the physical stimulation provided by shock waves could involve recruitment and further differentiation of stem cells [14-17].

Intriguingly, human Tendon-derived Stem/Progenitor cells (hTSPCs) were efficiently isolated from tendons of various species, including human hamstring tendons [18-20]. These local progenitor cells showed a self-renewal capacity, the potential to differentiate along an assortment of mature cell lineages -including tenocytes [21]- and are supposed to enhance tendon repair in several models [22-24]. Therefore, it has been recently proposed a function of resident stem cells in promoting tendon repair [1].

In light of those observations, we aimed to investigate the possible effects of shock wave exposure on hTSPCs explanted from healthy semitendinosus or ruptured Achilles tendons. In order to assess the ESWT putative burden on cell differentiation, we treated our cultures at the optimal dose accurately selected in previous investigations [11-13]. Our data show that this treatment significantly promotes the *in vitro*-induced hTSPCs differentiation.

## RESULTS

### ST and AT cells share similar characteristics

We established 10 different primary cultures of human tenocytes, 5 derived from healthy semitendinosus (ST) and 5 from ruptured Achilles (AT) tendons (Table 1), as previously described [12]. Human tendon-derived cells, both from ST or AT, were plastic-adherent and after 2 weeks of culture were subjected to cell characterization.

**Table:** ST and AT cells share similar characteristics

Healthy ST Cells				
Specimen Code	Age	*Fibroblast-like* phenotype	Vimentin	G2/M
**ST1**	16	+++	+++	11,4
**ST2**	19	+++	+++	7,5
**ST3**	17	+++	+++	10,5
**ST4**	45	+++	+++	11,2
**ST5**	40	++	+++	11,1
**Mean**	**27,4**			**10,34**

Ruptured AT Cells				
Specimen Code	Age	*Fibroblast-like* phenotype	Vimentin	G2/M
**AT1**	19	++	+++	9,1
**AT2**	25	+++	+++	12,1
**AT3**	41	++	+++	8,7
**AT4**	56	+++	+++	12,5
**AT5**	29	++	+++	16,9
Mean	**34**			**11,86**

**Figure 1:**
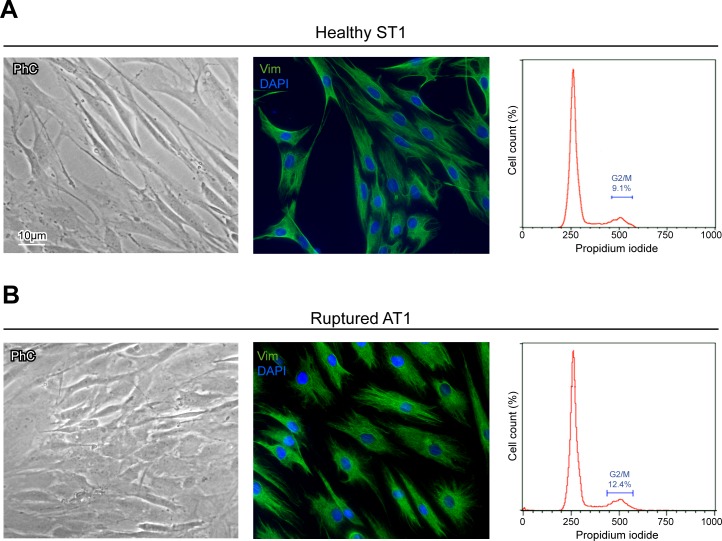
Culture cell characterization shows no significant differences in ST and AT cells Morphological analysis by phase contrast microscopy, vimentin evaluation by immunofluorescence and histograms plots of cell cycle distribution by flow cytometry in ST1 **A.** and AT1 **B.** cultures, representative of healthy and ruptured human tendon-derived cells. Nuclei are stained with DAPI. Bar 10 μm.

In both ST and AT cultures, phase contrast microscopy shows heterogeneous cell morphology, consisting in two main cellular patterns, as expected [1, 11, 18, 20, 25]. A prevalent *fibroblast-like* phenotype, spread on the plate and characterized by elongated cell bodies with thin processes, alternated with a minority of enlarged *tenoblast-like* cells (Figure 1), which become progressively more represented when cultures was reaching confluence (data not shown), as previously reported [11]. No significant morphological differences were detected between ST1-5 and AT1-5 cells (Table 1).

In order to evaluate possible differences in ST (ST1-5) and AT (AT1-5) cells, vimentin, a mesenchymal cell marker that labels cytoskeleton intermediate filaments, was then evaluated by immunofluorescence (Figure 1). A positive staining of perinuclear cytoplasmic bundles of filaments confirmed unequivocally the mesenchymal origin of all our cultures and excluded possible derangement of the cells (Table 1).

To evaluate the growth rate of ST (ST1-5) and AT (AT1-5) cells, we further analyzed the cell cycle distribution by flow cytometry (Table 1 and Figure 1). Results in Table 1 showed that both types of cultures were characterized by a similar mean percentage of cells in G2/M phase (10,34 in healthy ST1-5 and 11,86 in ruptured AT1-5), indicating the presence of proliferating cells in all our samples.

### Clonogenic potential is observed only on healthy human semitendinosus-derived cells

**Figure 2:**
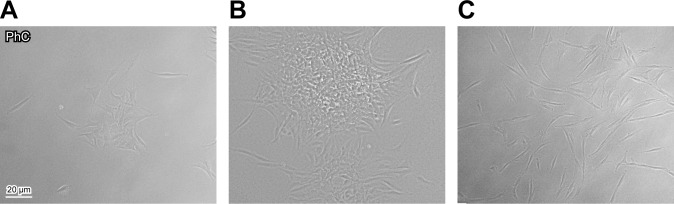
Cloning of tendon-derived cells Cloning of ST1, representative of healthy ST-derived cells, one **A.** and two **B.** weeks after plating. **C.** Uncloned AT1, representative of cells derived from ruptured AT, 12 days after plating. Bar: 20 μm.

Because stem cells typically display clonogenic potential, we tried to clone all our 10 primary cultures. We observed that they started to proliferate after few days of quiescence. Clonogenic cultures were generated by diluting suspension (1cell/μl) (as previously described [18, 19]) and they appeared heterogeneous in size and cell density. Therefore -according to previous authors [26]- we considered clones only those with diameter >2mm. In our samples, clonogenic potential seemed to be independent on tendon-derived patient age, but exclusive of ST cells (ranging from 16 to 45 years old, see Table 1): in fact, four out of five cultures derived from healthy ST were cloned and named ST1-4/C (Figure 2A-2B), but none of the cultures from ruptured AT could be cloned (Figure 2C).

### Expression of the differentiation marker alpha-smooth muscle actin (α-SMA) is significantly enhanced in uncloned AT cells

Considering that several authors reported a depletion of stem cell pool and a limited differentiation potential due to aging of the donors, for this part of the study we selected 6 cultures (ST1-2, the corresponding clones ST1-2/C and the uncloned AT1-2) derived from the youngest donors, ranging from 17 to 25 years old [1, 25, 27, 28].

**Figure 3:**
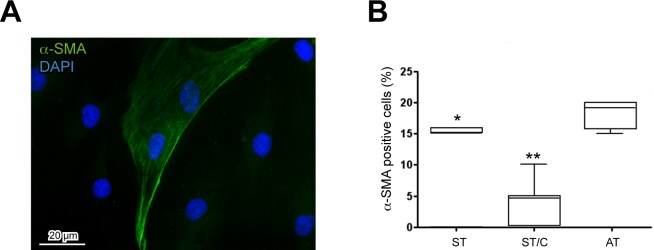
Expression of the differentiation marker alpha-smooth muscle actin (α-SMA) in healthy ST- and ruptured AT-derived cells **A.** The α-SMA signal is present in intracellular filaments situated at the cytoplasmic periphery of all the tendon-derived cells, here represented by AT1. Nuclei are stained with DAPI. Bar 20μm. **B.** Quantitative analysis of a-SMA-positive cells in AT, ST and ST/C cells reveals a significant increase in ruptured AT compared to healthy ST cells (Mann-Whitney: *p < 0.05 and **p < 0.001 *vs* ST and ST/C respectively). The data are represented with the Tukey box-and-whisker plot; the values are expressed as median ± Interquartile Ranges (IR) from three independent experiments.

First we evaluated by immunofluorescence the expression of α-SMA, which is a differentiation marker for activated tenocytes, as previously suggested [29], showing a signal localized in intracellular filaments, also organized in bundles, situated in the peripheral areas of the cytoplasm, (as shown in Figure 3A). Quantitative analysis of α-SMA-positive cells revealed an increase of differentiation in uncloned AT, especially when compared to cloned ST/C, and to a lesser extent to uncloned ST cells (Figure 3B), suggesting that these different types of cultures are not at the same stage of differentiation.

### ST and AT cultures express stem cell-related surface markers

In order to better characterize the previously selected cultures (ST1-2, ST1-2/C and AT1-2), the expression of typical surface stem cell markers was then evaluated by flow cytometry. Because no single marker can certainly identify human tendon-derived stem or progenitor cells [18], a panel of surface antigens was examined and shown in Table 2.

**Table:** FACS analysis of typical surface stem cell marker expression in ST and AT cells

Surface stem cell markers expression (%) in ST and AT-derived cells					
Specimen Code	CD146	CD44	CD90.2	CD105	CD34
**ST1**	36	95,8	99,6	99,6	3
**ST2**	48,2	99	99,9	97,8	4,4
**ST1/C**	96,2	93,7	99,9	99,7	1
**ST2/C**	60,5	91,3	99,2	95,8	2
**AT1**	8,45	96,2	99, 5	99,6	9,6
**AT2**	9,5	86,7	88	86,2	4,4

**Figure 4:**
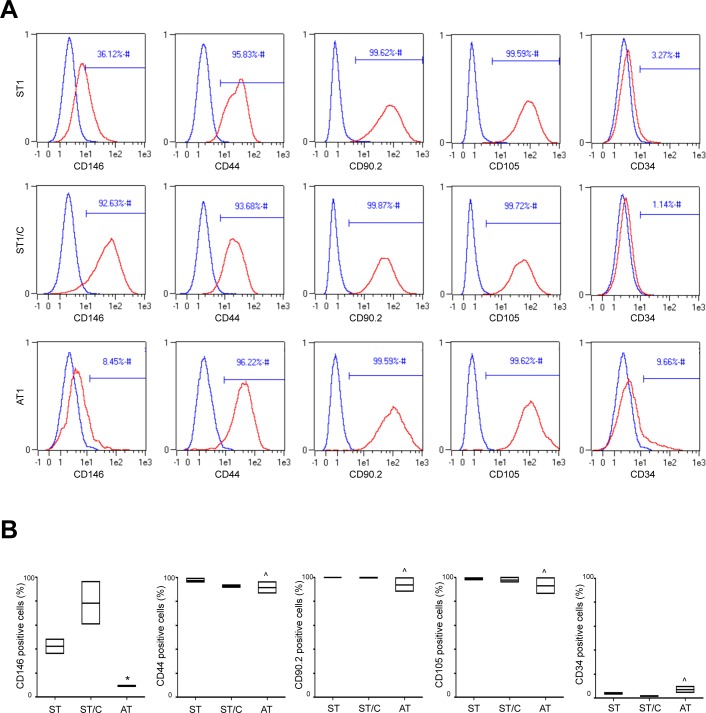
ST, ST/C and AT cells express typical surface stem cell markers Typical cell surface markers are analyzed by flow cytometry in ST1-2, ST1-2/C and AT1-2 cells. **A.** The figure shows a representative plots of ST1, ST1/C and AT1 cell cultures. In histograms, the curves of surface stem cells markers (in red) are overlayed with the unmarked cells (in blue). **B.** The cumulative data of stem cell markers are represented with the floating bars; the values are expressed as median ± Interquartile Ranges (IR) from three independent experiments (Mann-Whitney: *p < 0.05 *vs* ST and ST/C; ^*p*=not significant *vs* ST and ST/C).

FACS analysis showed pretty similar profiles of cell surface markers in cells explanted from healthy (ST) or ruptured (AT) tendons. All cultures revealed positive expression for the fibroblast marker CD90.2 (88 to 99,9%) and for the putative mesenchymal stem markers CD44 (86,7 to 99%) and CD105 (86,2 to 99,9%), and were mainly negative for the hematopoietic stem cell marker CD34 (1 to 9,6%). Nevertheless, a heterogeneous expression for the stem cell marker CD146, which is directly linked to multipotency [30], was observed. As shown in Figure 4, CD146 values were significantly higher in cells derived from healthy, ranging from 36 to 48,2% in ST1-2 and from 60,5 to 96,2% in corresponding clones ST1-2/C, compared to cells derived from ruptured samples, ranging in AT1-2 from 8,45 to 9,5% (Figure 4A-4B).

### ESWT enhances proliferation and differentiation of ST and AT cells

Firstly, to evaluate the proliferative rate of our cultures in response to ESWT, we performed a Ki67 analysis -a nuclear marker of cycling cells- showing significant differences between tendon-derived cells after and before cloning, as well as from those derived from healthy ST compared to pathological AT tendons (Figure supplemental 1A). Quantitative analysis of the percentage of Ki67^+^ cells revealed an ESWT-mediated proliferative effect on ST1-2, ST1-2/C, and AT1-2 cultures compared with control (*p* < 0.05). Interestingly, the shock wave-mediated proliferative effects were evident 12 days after the treatment -which is consistent with our previous data [11], especially in cloned (ST/C) compared to uncloned (ST and AT) cells and in ruptured (AT) versus healthy (ST) tendon-derived cells (Figure supplemental 1B).

Then the effect of ESWT on adipogenic, chondrogenic and osteogenic differentiation was evaluated using specific differentiation culture media (DM) on the previously selected 6 cultures.

Cell morphology was first assessed -by light microscopy and for few days- during the initial growth. Similarly to the results reported in Figure 1, cultured cells continued to be relatively heterogeneous, showing the same appearance described above. No significant morphological differences were observed in ESWT-treated cultures derived from healthy (ST1-2, ST1-2/C) or ruptured tendons (AT1-2) (data not shown).

**Figure 5:**
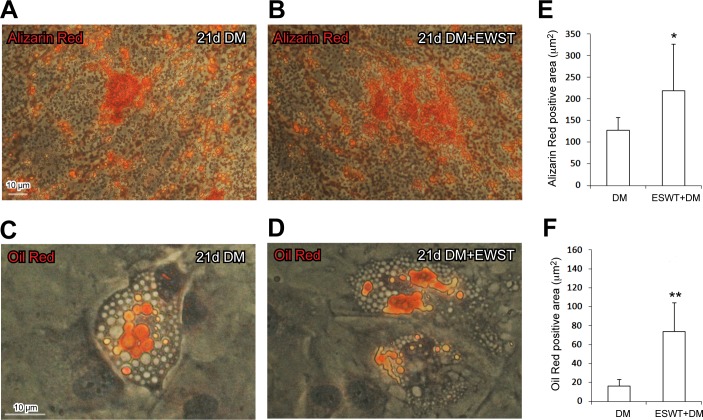
ESWT significantly increases the expression of differentiation markers in cells grown in specific differentiation media (DM) The clone ST1/C, representative of hTSPCs is shown for the cytochemical analysis in Figure 5A-5D (Bar: 10μm). Cytochemical analysis -by light microscopy- 21 days of DM cell-culture in ESWT pre-treated cells **B.**, **D.** compared to DM cell culture in untreated cells **A.**, **C.** Results expressed in Figure 5E-F represent the mean value ± SD measured on 10 digital frames for each culture, for osteogenic (5A-5B, Alizarin Red depots, *p < 0.05 *vs* untreated) as well as adipogenic (5C-5D, Oil Red droplets, **p < 0.0005 *vs* untreated) differentiation.

The ESWT-mediated response on differentiation was further evaluated, upon 21 days in specific differentiation media (DM), by cytochemical evaluation of differentiation markers performed by the detection of Oil Red positive droplets in adipogenic medium cultured cells and Alizarin Red positive calcium-rich depots in osteogenic medium cultured cells. We never detected neither Oil Red positive droplets nor Alizarin Red calcium depots in cells cultured in DMEM basal medium. ESWT significantly increased the differentiation of ST, ST/C or AT-derived cells, grown in specific differentiation media (DM) (Figure 5A-5F). As shown in Figure 5, ESWT improved the osteogenic (detected by Alizarin Red depots) and the adipogenic (detected by Oil Red droplets) differentiation. No significant cytochemical differences were observed in cloned ST/C compared to uncloned ST1-2 and AT1-2 cultures, as well as in ST versus AT derived cells, grown in DM either pre-treated with ESWT or untreated (data not shown).

**Figure 6:**
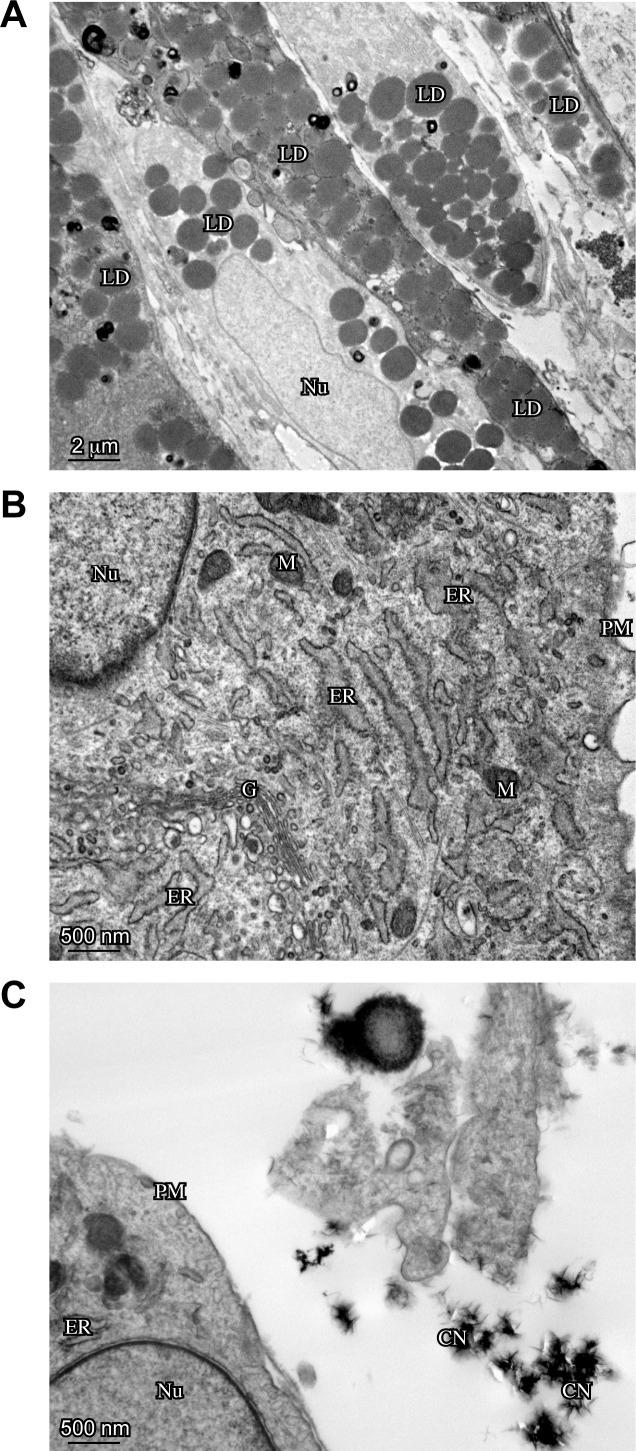
Ultrastructural analysis -by TEM- of ESWT-treated hTSPCs The clone ST1/C, representative of hTSPCs is shown for the ultrastructural analysis. **A.** Electron microscopy analysis shows the presence of lipid droplets (LD) in adipogenic medium-cultured hTSPCs (Bar: 2μm). **B.** hTSPCs grown in chondrogenic medium shows abundant rough Endoplasmic Reticulum (ER) and prominent Golgi apparatus (G) (Bar: 500nm). **C.** Calcium depots (CN: Centers of initial Nucleation) are present in osteogenic medium-cultured hTSPCs (Bar: 500nm). Mitochondrion (M), Nucleus (N), Plasma Membrane (PM).

Moreover, in order to deeper investigate the cell morphology, an ultrastructural analysis by transmission electron microscopy (TEM) was performed. The substantial heterogeneity in cell appearance previously documented by light microscopy was confirmed by TEM analysis of ST, ST/C and AT cells, before or after ESWT: in fact, while most of them showed thin plasma membrane protrusions, others were characterized by an enlarged morphology lacking cell processes. Lipid droplets were clearly detectable in adipogenic medium-cultured cells (Figure 6A), whereas calcium depots are visible in nearby osteogenic medium-cultured cells (Figure 6C). The cytoplasm of the cells cultured in chondrogenic medium was heterogeneously filled with stack of regularly-aligned cisternae of rough endoplasmic reticulum and prominent Golgi apparatus, indicating their secretory activity (Figure 6B).

According to the previous analysis, again no significant morphological differences were observed in ST, ST/C or AT cultures, either before or after ESWT. In addition, TEM confirmed that ESWT enhances the *in vitro*-induced cell differentiation, either to adipogenic or osteogenic lineage (data not shown).

**Figure 7:**
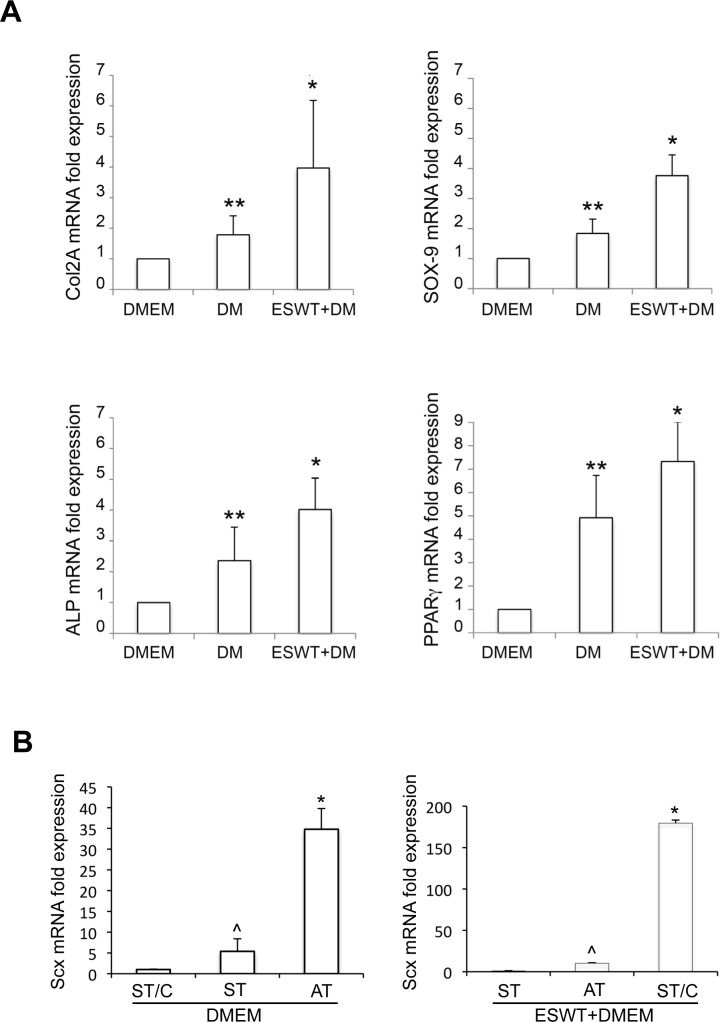
Molecular analysis of typical differentiation markers -by RT-PCR- of ESWT-treated hTSPCs **A.** mRNA expression of COL2A, SOX9 (chondrogenic differentiation), ALP (osteogenic differentiation), and PPARγ (adipogenic differentiation) was evaluated after 21 days of culture in differentiation media (DM) following or not ESWT in ST/C, ST and AT cells; (*p < 0.01: ESWT+DM *vs* DM; **p < 0.05: DM *vs* DMEM). Corresponding gene expression in cells cultured in DMEM was used as negative control and values were normalized to DMEM, set to 1. **B.** mRNA expression of Scx (early differentiation marker) was evaluated after 12 days of culture in DMEM basal medium ±ESWT. In untreated samples, values were normalized to ST/C cells, set to 1 (*p < 0.005: AT *vs* ST and ST/C; ^*p*=not significant: ST *vs* ST/C). In ESWT-treated samples, values were normalized to ST cells, set to 1 (*p < 0.0005: ST/C *vs* AT and ST; ^*p*=not significant: AT *vs* ST).

The effects of ESWT on differentiation were then assessed by RT-PCR -on the 6 cultures as above (ST1-2, ST1-2/C and AT1-2)- to determine the expression of specific genes activated in response to differentiative stimuli. COL2A and SOX9 expression was selected to analyze the chondrogenic lineage, ALP, BGLAP and RUNX2 for the osteogenic lineage, and PPARγ for the adipogenic differentiation. Results showed that, after 21 days of culture, there was an ESWT-mediated significant up-regulation in the expression of COL2A and SOX9 for chondrogenic differentiation, of ALP for osteogenic and PPARγ for adipogenic differentiation in all types of cells (*p* < 0.05: ESWT+DM *vs* DM alone), confirming the cytochemical observations (Figure 7A). In contrast, the osteogenic markers BGLAP and RUNX2 were not significantly different. The gene expression profiles of cells cultured in DMEM was used as negative control. Again, no significant differences were detectable in cultures derived from healthy semitendinosus (ST and ST/C) or from ruptured Achilles (AT) tendon explants (data not shown).

Further molecular analysis to control ESWT-mediated effects was performed in ST, ST/C or AT cultures grown 12 days in basal medium, measuring mRNA transcript levels of the early differentiation tenocyte marker Scleraxis (Scx) (Figure 7B) [31, 32]. In untreated samples, Scx expression was significantly higher in ruptured AT-derived compared to healthy ST-derived cells. In ESWT-treated samples, Scx levels were significantly increased in ST/C, compared to uncloned ST and AT cultures.

In this work we designated both types of cultures as human Tendon-derived Stem/Progenitor cells (hTSPCs), according with previous report [18].

## DISCUSSION

In this study, we show that ESWT is able to accelerate the *in vitro*-induced differentiation of human primary cultured Tendon Stem/Progenitor cells. In a previous study, we demonstrated that ESWT increases the functional activities of tenocytes explanted from human tendons -including proliferation, migration and collagen synthesis- without modifying cell viability [11-13]. Moreover we also showed that cultures derived from ruptured tendons were more susceptible to shock wave-mediated activation, indicating a possible recruitment of undifferentiated progenitors in the site of injury [12, 13]. Here we confirm that ESWT induces cell proliferation, although our cultures are not at the same stage of differentiation, and are characterized by a heterogeneous natural differentiation potential, probably initiated by tendon injuries [12, 13]. Therefore, according with other reports [14, 15, 33], the clinical benefits of ESWT can be explained by mechanical stress promoting the *in vivo* regenerative capacity of the tissue.

However it is widely accepted that physiological repair after tendon damage is pretty limited [34]. As recently suggested, human stem cells are considered a suitable tool for the management of human chronic tendinopathies, albeit their effective contribution to tendon regeneration *in vivo* is still matter of discussion [2, 5, 15, 19, 24, 35, 36, 37]. Cultures of tendon-derived stem cells were established and several reports investigated strategies to enhance differentiation into tenocytic-like lineage [18, 38, 39]. The International Society for Cellular Therapy (ISCT) proposed the minimal criteria to define human mesenchymal stem cells (MSCs), including the capacity to be plastic-adherent, proliferate, form colonies, express typical CD markers and differentiate in vitro to osteoblasts, adipocytes and chondroblasts [40].

In the present work, we describe these capacities in a variable percentage of healthy semitendinosus (ST) and ruptured Achilles (AT) human tendon-derived cells that we designated as human Tendon-derived Stem/Progenitor Cells (hTSPCs), according with previous report [18]. Since we published that the *in vitro* biological behaviour of cells explanted from healthy compared to ruptured tendons was significantly different [12], possible variations also in establishing human tendon-derived stem cells from those sources could be expected. Although proliferative capacity and multipotency are detectable in all our cultures, the clonogenicity potential is exclusive of healthy ST-, whereas ruptured AT-derived cells grow separately and do not form colonies. Interestingly, the expression of α-SMA and Scx tenocytic differentiation markers is significantly enhanced in uncloned AT cells, whereas the stem cell-related surface marker CD146, probably linked to multipotency [30], was significantly higher in ST cells. Therefore, although Zhang and colleagues showed a different ability to form colonies in tendon progenitor cells derived from patellar and Achilles tendons of rabbits [19], we previously published that our cell characterization showed no significant differences between Achilles and semitendinosus tendon-derived cultures [12]. For this reason, we propose that the differences observed in our samples may be amenable to the damage occurred in human AT and its physiological attempt to repair the injury, although we cannot definitively exclude that they are due to their different origin.

It is likely that a variable amount of resident hTSPCs started a differentiative program to regenerate the tissue. Intriguingly, even though uncloned ruptured AT cells already started a differentiation program toward the tenocytic lineage, all hTSPCs cultures partially maintain a multidifferentiative potential, as ascertained by their capacity to differentiate *in vitro*. Although the physiological repair of tendons remains difficult, it is conceivable that our tendon-derived cells can be facilitated by specific treatments, such as ESWT, to differentiate toward tenocytic-like lineage.

Consistent with this hypothesis, we show for the first time that ESWT significantly enhances hTSPCs-induced differentiation, as indicated by cytochemical and molecular findings. Our results are mainly in agreement with previous reports performed *in vitro* on equine adipose tissue-derived stem cells [41], rat and human adipose-derived stem cells [17, 42] and human bone marrow stromal cells [15, 16]. Surprisingly, no significant differences in the appearance and behavior of healthy ST and ST/C compared to AT cells was induced by ESWT, implying that the different amount of hTSPCs present in both types of explants share similar characteristics. Therefore we suppose that in our cultures may coexist cellular elements bearing different degrees of differentiation, all being sensitive to shock wave stimulation.

Taken together, previous reports and the present observations seem to corroborate the long-held idea that ESWT may enhance recruitment, proliferation and differentiation of tissue-derived stem cells [15, 17, 41]. We propose that such treatment, applied on injured tendons, is able to accelerate stem cell differentiation -probably through the release of different cytokines and growth factors- mainly towards tenocytic lineage, contributing to tendon repair.

Further investigations will certainly help to better clarify that ESWT clinical benefits are amenable to mechanisms triggering tendon healing promoted by resident stem cells recruitment.

## MATERIALS AND METHODS

### Patients

Adult human tendon-derived cells were explanted from 5 healthy semitendinosus (ST) tendons of patients affected by Anterior Cruciate Ligament (ACL) rupture and subjected to ligament reconstruction by ST, and 5 from patients affected by Achilles (AT) tendon ruptures. The average age of the patients was 31 ± 15 years.

The Institutional Review Board of “Sapienza” University and Sant'Andrea Hospital approved the study protocol, and all patients gave their written informed consent to the experimental study.

### Isolation, culture and cloning of tendon-derived cells

Freshly isolated tendon-derived cells, from ST or AT, were seeded in 24well/plates (or T25 plates) and cultured around 3 weeks in conventional medium (DMEM with 10% FBS), as described [11-13]. In order to form colonies, cells were cultured 8-10 days in Tissue Culture Dishes (Corning 100×20mm) after a diluting suspension to 1cell/μl, as previously described [18, 19]. Single cell colonies, when clearly detectable and more than 2mm in diameter, were detached by local application of trypsin, transferred to T25 flasks for further culture, rapidly proliferated reaching around 80% confluence and finally used for the following experiments. Also cells that were not able to form colonies were similarly detached and cultured, and used for the following experiments.

### Immunofluorescence

Cells grown on coverslips were fixed with 4 % paraformaldehyde followed by treatment with 0.1 M glycine for 20 min at 25°C and with 0.1 % Triton X-100 for additional 5 min at 25°C to allow permeabilization.

Cells were then incubated alternatively with the following primary antibodies: the anti-vimentin (1:50 in PBS; Dako, Glostrup, Denmark) and alpha-smooth muscle actin (α-SMA) (1:50 in PBS; Dako) monoclonal antibodies and the rabbit polyclonal antibody anti-Ki67 (1:50; Zymed Laboratories; San Francisco, CA, USA). The primary antibodies were visualized, after appropriate washing with PBS, by using goat anti-mouse IgG-FITC (1:50 in PBS; Cappel Research Products, Durham, NC, USA), for 30 min at 25°C and the goat anti-rabbit IgG-Texas Red (1:200; Jackson Immunoresearch Laboratories, West Grove, PA, USA). Nuclei were stained with 4′-6′-diamido-2-phenylindole dihydrochloride (DAPI) (1:10,000 in PBS, Sigma Chemical Co., St. Louis, MO, USA). Coverslips were finally mounted with Mowiol in PBS for observation. Fluorescence signals were analyzed by conventional fluorescence or by scanning cells in series of 0.5 μm sequential sections with an ApoTome System (Zeiss, Oberkochen, Germany) connected with an Axiovert 200 inverted microscope (Zeiss); image analysis was then performed by the Axiovision software (Zeiss). The percentage of vimentin, α-SMA and Ki67-positive cells was analyzed counting for each treatment a total of 500 cells, observed in ten microscopic fields randomly taken from three different experiments. Results have been expressed as mean values ± Standard Deviation (SD).

### Flow cytometry analysis

For the analysis of cell cycle, each culture was plated sparsely at P1 so that it did not touch each other and did not reach contact inhibition. Cells were trypsinized, pelleted and resuspended in 70% ethanol in PBS, and stored at 4°C overnight. Then they were washed with PBS, resuspended in propidium iodide (PI) staining solution (50 mg/ml) and RNAse A (100 Kunitz/ml) (Miltenyi Biotec GmbH, Bergisch Gladbach, Germany) and incubated in the dark for 40 minutes at room temperature.

For the surface antigen expression, the MoAbs used were: CD146-PE, human (Miltenyi Biotec GmbH), CD90.2-APC (Miltenyi Biotec GmbH), CD44-FITC (Becton, Dickinson and Company, Franklin Lakes, New Jersey), CD105-APC (Miltenyi Biotec GmbH) and CD34-PE (Miltenyi Biotec GmbH). Negative control was performed using the HaCaT, spontaneously immortalized human keratinocyte cell line.

For this assay the cells were harvested and stained according to the manufacturer instructions. Each measurement contained at leats 30,000 cells collected with MACSQuant® Analyzer flow cytometer (Miltenyi Biotec GmbH) and analysed with MACSQuantify® software (Miltenyi Biotec GmbH).

### *In vitro* differentiation and proliferation of tendon-derived cells

Cellular samples were then passed and further cultured in specific differentiation media for osteocytes, chondrocytes or adipocytes. Differentiation of each sample was performed using specific media to promote osteogenic, chondrogenic and adipogenic differentiation, respectively. Cells were cultured for osteogenic differentiation in hMSC Osteogenic Diff BulletKit (LONZA, Basel Switzerland), for chondrogenic differentiation in hMSC Chondrogenic Diff BulletKit and TGF-β3 for hMSC Chondro (LONZA, Basel Switzerland), and for adipogenic differentiation in hMSC Adipogenic Diff BulletKit (LONZA, Basel Switzerland), as previously described [43].

In order to evaluate their possible influence on in vitro cell differentiation, cultures were subjected to ESWT as recently described [11-13], using an electromagnetic shock wave generator MODULITH® SLK (STORZ MEDICAL AG; Tägerwilen, Switzerland), at a dose of shock waves 0.14 mJ/mm^2^ energy level and 1000 impulses as previously described [11-13], whereas the control group was maintained in the same culture conditions, consisting in conventional or differentiation media, without previous shock wave exposure. Shock wave intensity and energy levels were previously selected [11-13] in order to maximize the therapeutical effects without significantly decrease the cell viability.

A cryogenic vial (Corning Incorporated, NY, USA) containing a cell suspension with 1.10^6^ cells/ml was placed on shock wave generator with a coupling gel (Aquasonic 100; Parker Laboratories, Fairfield, New Jersey, USA) to minimize the loss of shock wave energy at the interface between the head of the device and the cryovial. The tube was placed exactly in the focus of the application pad under ultrasonographic control.

In particular, cells destined to morphological/cytochemical and molecular analysis were grown 2-3 days in the Differentiation Media (DM), detached and exposed to ESWT, and further cultured 3 weeks in DM. Controls were grown 3 weeks in conventional or differentiation media, without shock wave exposure. According with our previous reports [11, 12], cells evaluated for Ki67 proliferation and Scx early differentiation marker were grown 12 days after ESWT in conventional medium, whereas controls were grown the same days without previous shock wave exposure.

### Morphological analysis

The morphological analysis of human tendon-derived cells -either from healthy ST or ruptured AT- was performed during cell growth in specific differentiation media, following ESWT, using an Axiovert 200 inverted microscope (Zeiss, Oberkochen, Germany) equipped with Axiovision image analysis system (Zeiss).

### Transmission electron microscopy

Human primary cultures, isolated and treated as above, were fixed with 2% glutaraldehyde in PBS at 4-C. Samples were postfixed in 1% osmium tetroxide in veronal acetate buffer (pH 7.4) for 1 hour at 25°C, stained with uranyl acetate (5 mg/ml) for 1 hour at 25°C, dehydrated in acetone and embedded in Epon 812 (EMbed 812, Electron Microscopy Science, Hatfield, PA, USA). Ultrathin sections, unstained or poststained with uranyl acetate and lead hydroxide, were examined under a Morgagni 268D transmission electron microscope (FEI, Hillsboro, OR, USA), equipped with a Mega View II charge-coupled device camera (SIS, Soft Imaging System GmbH, Munster, Germany) and analyzed with AnalySIS software (SIS, Soft Imaging System GmbH, Munster, Germany).

### Quantitative evaluation of osteogenic and adipogenic differentiation markers

The evaluation of ESWT potential to influence differentiation of tendon-derive cells grown in specific media (as described earlier) was performed by cytochemical staining of typical differentiation markers, Oil Red for adipocytes and Alizarin Red for osteocytes. In particular, after 3 weeks of culture, ESWT treated/untreated cells were stained using Oil Red O assay kit (Millipore), as indicator of intracellular lipid droplets in adipocytes or Alizarin Red S assay kit (Millipore) as indicator of mineral depots in osteocytes, following manufacturer's instructions. Digital images were collected by a Axiocam (Zeiss) CCD camera and analyzed by the Axiovision (Zeiss) software. Background and positive levels of staining were defined in control slides. For Oil Red and Alizarin Red, samples -observed at a final magnification of 320X- were scanned for the whole length and at least 10 digital frames were randomly taken for each conditions. Values are expressed as percentage of positive area/total frame area ± Standard Deviation (SD).

### Molecular analysis of chondrogenic, osteogenic, adipogenic and tenocytic differentiation

The ESWT potential to influence differentiation of tendon-derived cells grown in osteogenic, chondrogenic and adipogenic media was further tested, determining by qRT-PCR the expression of specific genes activated in response to differentiative stimuli. Oligonucleotide primers for target genes and for the housekeeping gene were chosen with the assistance of the Oligo 5.0 computer program (National Biosciences, Plymouth, MN) and purchased from Invitrogen. The following primers were used: for alkaline phosphatase, ALP target gene: 5′-CAAGGACGCTGGGAAATCTGT-3′ (sense), 5′-AGGGGGCATCTCGTTGTCT-3′ (anti-sense); for Bone Gamma-Carboxyglutamate (Gla) Protein, BGLAP target gene: 5′-CCTCACACTCCTCGCCCTAT-3′ (sense), 5′-GCTTGGACACAAAGGCTGC-3′ (anti-sense); for the Run-related transcription factor 2, RUNX2 target gene: 5′-GAACCCAGAAGGCACAGACA-3′ (sense), 5′-GCGGGACACCTACTCTCATAC-3′ (anti-sense); for the transcription factor, SOX9 target gene: 5′-CCCCCAACGCCATCTTCAA-3′ (sense), 5′-CTGGGATTGCCCCGAGTG-3′ (anti-sense); for the type II collagen, COL2A target gene: 5′-ACACTGGGACTGTCCTCTGCGA-3′ (sense), 5′-CCTTTGGTCCTGGTTGCCCACTG-3′ (anti-sense); for the peroxisome proliferator activator receptor gamma, PPARγ target gene: 5′-GGAAAGACAACAGACAAATCACCAT-3′ (sense), 5′-ACCTCTTTGCTCTGCTCCTG-3′ (anti-sense); for the transcription factor scleraxis A, SCXA target gene: 5′-CGAGCGAGACCGCACCAACA-3′ (sense), 5′-CGTTGCCCAGGTGCGAGATGTAG-3′ (anti-sense); for the 18S rRNA housekeeping gene: 5′-AACCAACCCGGTCAGCCCCT-3′ (sense), 5′-TTCGAATGGGTCGTCGCCGC-3′ (antisense). For each primer pair, we performed no-template control and noreverse-transcriptase control (RT negative) assays, which produced negligible signals. RNA was extracted using the TRIzol method (Invitrogen, Carlsbad, CA) according to manufacturer's instructions and eluted with 0.1% diethylpyrocarbonate (DEPC)-treated water. Total RNA concentration was quantitated by spectrophotometry and the quality was assessed by measuring the optical density ratio at 260/280 nm. RNA samples were stored at −80°C. After denaturation in DEPC-treated water at 70°C for 10 min, 1 μg of total RNA was used to reverse transcription using iScriptTM cDNA synthesis kit (Bio-Rad) according to manufacturer's instructions. Real-time PCR was performed using the iCycler Real-Time Detection System (iQ5 Bio-Rad) with optimized PCR conditions. The reaction was carried out in 96-well plate using iQ SYBR Green Supermix (Bio-Rad) adding forward and reverse primers for each gene and 1 μl of diluted template cDNA to a final reaction volume of 15 μl. All assays included a negative control and were replicated three times. The thermal cycling programme was performed as follows: an initial denaturation step at 95°C for 3 min, followed by 45 cycles at 95°C for 10 sec. and 60°C for 30 sec. Real-time quantitation was performed with the help of the iCycler IQ optical system software version 3.0 a (Bio-Rad), according to the manufacturer's manual. The relative expression of the housekeeping gene was used for standardizing the reaction. The comparative threshold cycle (Ct) method was applied to calculate the fold changes of expression compared to control cells. Results are reported as mean ± SD from three different experiments in triplicate.

### Statistical analysis

To compare variables that assume normal distribution, Student's T test was calculated. The values are expressed as mean ± SD or SE from three independent experiments.

To compare variables that do not assume Gaussian distribution, Mann-Whitney non-parametric test was used. The values are expressed as median ± Interquartile Ranges (IR) from three independent experiments. The data are represented with the floating bars or Tukey box-and-whisker plot; the central box represents the values from the lower to upper quartile (25th to 75th percentile), the middle line represents the median, and the horizontal lines represent the minimum and the maximum value of observation range.

*P* values <0.05 were assumed as statistically significant.

## SUPPLEMENTARY MATERIAL FIGURE


