# Supplementary material for: Extracorporeal Shock Wave Treatment (ESWT) enhances the in vitro-induced differentiation of human tendon-derived stem/progenitor cells (hTSPCs)
Source: Oncotarget. 2016 Jan 28;7(6):6410–23. doi: 10.18632/oncotarget.7064 (PMC4872723; doi:10.18632/oncotarget.7064)
Supplement: Supplementary file 1 [file oncotarget-07-6410-s001.pdf]

# Extracorporeal Shock Wave Treatment (ESWT) enhances the *in vitro*-induced differentiation of human tendon-derived stem/progenitor cells (hTSPCs)

## Supplementary Material

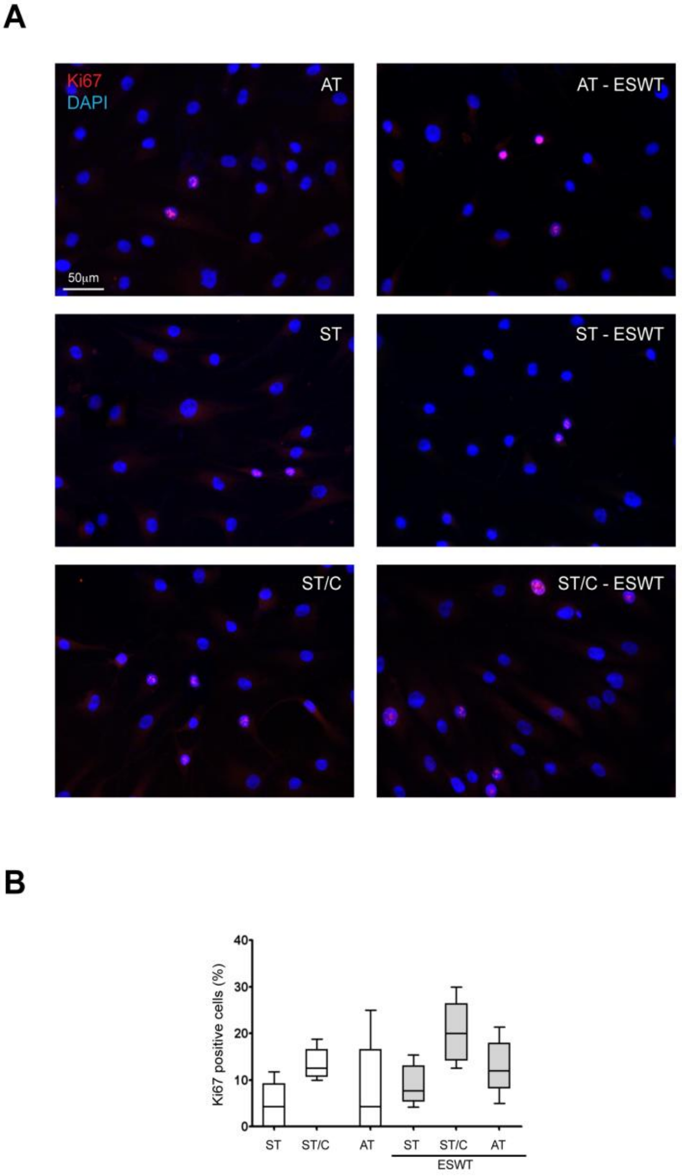


***Figure 1 Supplemental.*** **ESWT effects on proliferation of hTSPCs.**

(A) Immunolabeling with anti-Ki67 (red) was achieved on hTSPCs cultures at 12 days after shock wave exposure. Nuclei are stained with DAPI. Photomicrographs are representative of each culture. (B) Quantitative immunofluorescence analysis of proliferation induced by ESWT was performed. Bar 50 μm.
